# Spinal cord protection by epidural separation during vertebral cryoablation for metastatic spine disease: A proof-of-concept preclinical study

**DOI:** 10.1016/j.bas.2026.105975

**Published:** 2026-02-13

**Authors:** Takaaki Uto, Satoshi Kato, Noriaki Yokogawa, Takaki Shimizu, Motoya Kobayashi, Satoshi Nagatani, Masafumi Kawai, Yuji Ishino, Kazuhiro Nanpo, Megumu Kawai, Satoru Demura

**Affiliations:** Department of Orthopedic Surgery, Graduate School of Medical Sciences, Kanazawa University, Kanazawa, Ishikawa, Japan

**Keywords:** Spinal metastases, Cryoablation, Spinal cord injury, Epidural separation, Animal model

## Abstract

**Introduction:**

Cryoablation for spinal metastases is limited by the risk of cryogenic neural injury near the spinal cord. Physical separation between the vertebral body and dura may provide a thermal margin, but the distance–temperature relationship in the epidural space remains insufficiently defined.

**Research question:**

This exploratory study aimed to map the association between surgically created epidural separation distance and local thermal and neurophysiological changes during vertebral cryoablation in a canine model.

**Material and methods:**

In a non-randomized, unblinded, two-phase pilot study, we first performed *in vitro* phantom experiments (0-, 2-, and 5-mm air gaps). Subsequently, 12 beagle dogs were assigned to three groups (n = 4 each): 0-mm (control), 2-mm, or 5-mm epidural separation, followed by T13 vertebral cryoablation. Primary outcomes were the minimum epidural temperature at the ventral midline and 30-min compound muscle action potential (CMAP) amplitude recovery.

**Results:**

*In vitro*, greater separation distance was associated with significantly warmer target-point temperatures (*P* < .001). *In vivo*, a 5-mm separation produced warmer ventral midline epidural temperatures, which remained above a previously reported safety estimate of 4 °C, and greater CMAP recovery compared to a 2-mm separation (*P* = .024 and *P* = .028, respectively).

**Discussion and conclusion:**

In this proof-of-concept canine study, epidural separation distance was associated with ventral epidural temperature and short-term CMAP recovery during vertebral cryoablation. These findings do not establish a clinical safety threshold and require validation in clinically relevant models, including studies with survival and histologic assessment.

## Highlights

• *In vitro* and *in vivo* models evaluated epidural separation during cryoablation.
• Greater separation was associated with warmer ventral epidural temperatures.
• Separation distance was associated with improved short-term CMAP recovery.

## 1 Introduction

Percutaneous image-guided thermal ablation, including cryoablation, has seen expanding indications and increasing use in clinical practice for musculoskeletal metastatic disease, and percutaneous ablation techniques have become increasingly adopted as part of multidisciplinary care for osseous spine metastases (Sagoo et al., 2022). However, its application near the spinal cord remains limited by the risk of cryogenic neural injury (Tomasian et al., 2016). This limitation is clinically important because metastatic spinal tumors frequently involve the posterior vertebral body and the epidural space, where small safety margins can translate into neurological deficits and functional loss (Mut et al., 2005; Cole and Patchell, 2008). Although radiotherapy and analgesics remain standard for nonoperative cases, local progression and pain can persist, and aggressive surgery can carry substantial morbidity depending on the extent of resection (Laufer et al., 2013; Conti et al., 2019). Therefore, strategies that enable local tumor treatment while minimizing neural risk remain a priority.

In other organs, displacement techniques are widely used to reduce collateral thermal injury during ablation. Hydrodissection, gas insufflation, and related methods create a physical buffer that increases the distance between the ablation zone and vulnerable adjacent structures, thereby modifying local heat transfer and improving procedural safety margins (Tsoumakidou et al., 2011; Seager et al., 2021; Adebanjo et al., 2024). These principles suggest that controlled separation could also be relevant for vertebral cryoablation performed adjacent to the spinal cord.

However, applying this principle to the spine is not straightforward. The epidural space is confined and anatomically complex, which can make displacement techniques such as hydrodissection technically challenging and less predictable. Consequently, a key knowledge gap persists: in the epidural space, the separation distance required to meaningfully alter the local thermal environment and short-term neurophysiological responses has not been systematically quantified in any controlled model. Separation surgery was originally developed to create a corridor between the thecal sac and tumor to facilitate high-dose stereotactic radiosurgery, typically targeting a 2–3 mm margin (Khan et al., 2025; Echt et al., 2021). Whether a similar corridor provides a sufficient thermal margin for cryoablation, and what separation range should be considered for spinal applications, remain undefined (Kobayashi et al., 2024).

Accordingly, this proof-of-concept study aimed to map the association between epidural separation distance and (i) ventral midline epidural temperature and (ii) compound muscle action potential (CMAP) recovery during vertebral cryoablation, using an *in vitro* phantom for thermal context and an *in vivo* canine model. We evaluated 0 mm as a control condition, 2 mm to reflect the corridor commonly targeted for stereotactic radiosurgery, and 5 mm as an exploratory upper range to assess whether a larger separation could produce a more substantial thermal margin within the tested experimental setting.

## 2 Materials and methods

**Fig. 1:**
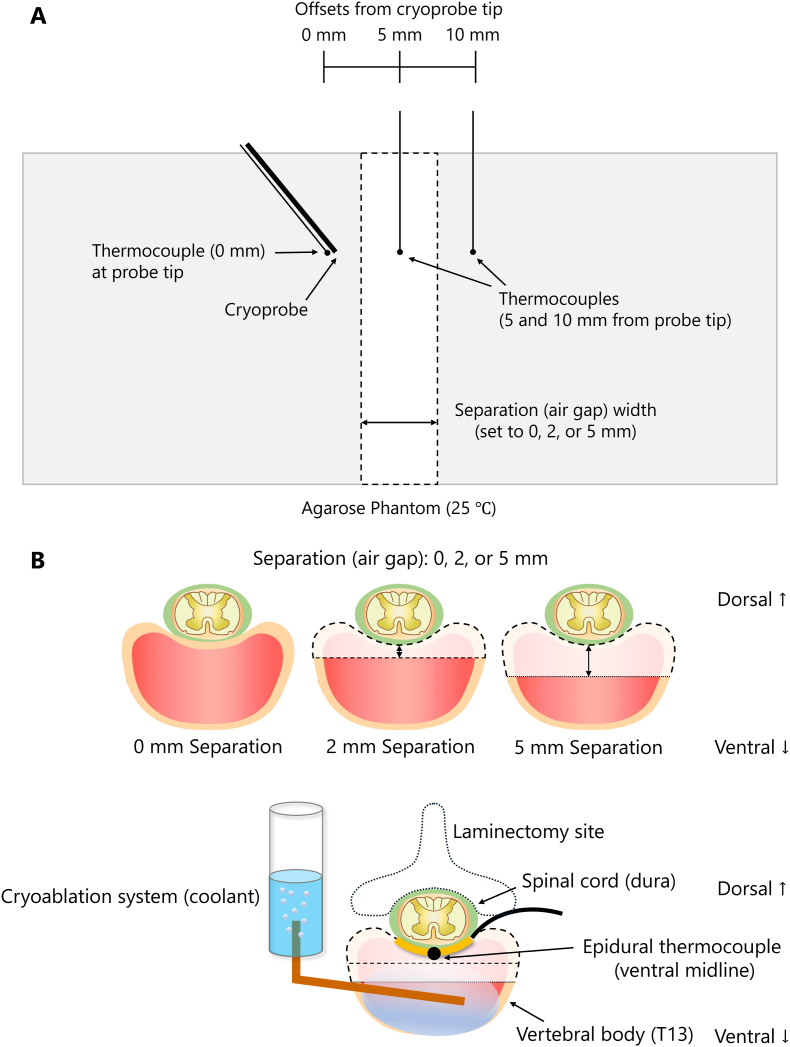
Schematic of the experimental setup. (A) *In vitro* phantom showing cryoprobe and thermocouples at the cryoprobe tip (0 mm), midpoint, and target point (10 mm). (B) *In vivo* canine setup showing the laminectomy at T13, cryoprobe trajectory into the vertebral body, and epidural thermocouple at the ventral midline.

Fig. 1 shows the experimental setup for the *in vitro* phantom and *in vivo* canine procedures.

### 2.1 *In vitro* phantom experiments

To investigate the thermal-insulating effect of a precisely controlled air-gap *in vitro*, we used a tissue-mimicking agarose–sucrose phantom. The phantom was designed to assess relative, distance-dependent thermal gradients under controlled conditions and was not intended to replicate absolute physiologic temperatures. For each 480-mL phantom, 9.6 g agarose (2% w/v) and 38.4 g sucrose (8% w/v) were dissolved in 455 mL of distilled water by microwave heating.

*Air-gap fabrication:* To create a well-defined separation, a removable acrylic plate (thickness of 2 or 5 mm) was temporarily positioned at the geometric center of the case, and the hot solution was poured on both sides. For the 0-mm condition, no plate was used. After gelation, the plate was removed, leaving a clean air gap.

*Sensor geometry (midpoint reference):* A T-type thermocouple was placed at the center of the air gap, which was defined as the midpoint. The cryoprobe (with a thermocouple attached to its tip) was inserted into one block, 5 mm from the midpoint. A third thermocouple (target point) was inserted into the opposing block 5 mm from the midpoint. Thus, as illustrated in Fig. 1A, the measurement locations were aligned along a line perpendicular to the gap at 0 mm (cryoprobe tip), 5 mm (midpoint), and 10 mm (target point), with all insertions performed at the same depth.

*Freezing protocol:* For each of the 10 replicates per condition (0-, 2-, and 5-mm), a new single-use phantom was prepared. After equilibrating to a stable room temperature (25 ± 1 °C), a single 10-min freeze was performed, and temperatures were recorded every 10 s.

### 2.2 Animal model and study design

This study was conducted in 12 beagle dogs to explore the association between epidural separation distance and epidural thermal and neurophysiological changes during vertebral cryoablation. Animals were assigned to three groups (n = 4 each): a 0-mm separation (control) group, a 2-mm group, and a 5-mm group. The study was designed as an exploratory, controlled, parallel-group experiment.

*Animals*: Twelve purpose-bred female beagle dogs (11–12 months old; 8.0–11.2 kg) were included. Beagle dogs were chosen because their thoracolumbar vertebral body size and spinal canal dimensions allow standardized cryoprobe placement and enable graded epidural separations under controlled experimental conditions. Animals were obtained from Kitayama Labes Co., Ltd. (Ina, Nagano, Japan). Physical examination and routine laboratory screening were performed to confirm their health before enrollment.

### 2.3 Sample size justification

The *in vivo* sample size of n = 4 per group was prespecified for this exploratory study based on a balance of feasibility, resource constraints, and ethical considerations (the 3Rs: replacement, reduction, and refinement) for a large-animal surgical model, rather than a formal statistical power calculation. The study was intended to generate initial effect estimates and hypothesis-supporting evidence for a novel procedure and not to provide definitive efficacy testing. We acknowledge that this small sample size limits the statistical power to detect subtle inter-group differences and increases the risk of Type II error.

### 2.4 Randomization and blinding

Randomization was not performed. The 12 animals were studied sequentially, with one animal per experimental day over 12 days. To standardize perioperative logistics and mitigate potential learning-curve effects, we used a prespecified fixed sequence of group assignments. The sequence comprised a 3-day block (0-mm, 5-mm, and 2-mm), which was repeated four times over the course of the study. We acknowledge that this fixed, unrandomized sequence may introduce systematic order or day-related confounding effects. Allocation concealment was not applicable.

Blinding of surgeons, intraoperative recorders, outcome assessors, or data analysts was not implemented. Masking was not feasible because both the separation procedure and real-time temperature monitoring display were integral to intraoperative safety and remained visible throughout data acquisition. This design limitation may have increased the risk of measurement and interpretation bias.

### 2.5 Housing and husbandry

Animals were housed in a climate-controlled facility (23 °C; 50% relative humidity) on a 12:12-h light–dark cycle with *ad libitum* access to food and water. Environmental enrichment was provided, and all dogs underwent a minimum 7-day acclimatization period.

### 2.6 Anesthesia and perioperative care

A standardized protocol was used for premedication (medetomidine/midazolam), induction, and maintenance of anesthesia (propofol infusion with 50% N_2_O/O_2_). Intraoperative monitoring included continuous electrocardiogram, SpO_2_, end-tidal carbon dioxide_2_, core temperature, and invasive arterial pressure. To avoid confounding outcomes from potential postoperative injuries, all procedures were terminal. At the end of data acquisition, animals were euthanized under deep anesthesia via secobarbital overdose followed by intravenous potassium chloride. Prespecified humane endpoints were monitored throughout the study but were not reached. Because motor pathway monitoring is sensitive to anesthetic agents, a uniform anesthetic regimen was used across all animals (propofol infusion with a fixed 50% N_2_O/O_2_ mixture). During CMAP recordings, physiological parameters were maintained within a stable range to minimize variability related to anesthesia and systemic physiology.

### 2.7 Separation surgery

After surgical preparation, a posterior midline longitudinal incision was made to expose the laminae from T11 to L2. A laminectomy was performed at T13 using a Medtronic Midas Rex high-speed drill system (Medtronic, Minneapolis, MN, USA), and the left proximal 13th rib was resected. In the 2-mm and 5-mm separation groups, the ventral aspect of the spinal canal was progressively excavated to create a separation space. The separation distance was confirmed intraoperatively by allowing free passage of the respective target burr (diameter: 2 mm or 5 mm) between the vertebral body and ventral dura mater. The 5-mm separation was achievable and confirmed in this controlled canine model; feasibility in cases of severe metastatic epidural compression was not evaluated. No spacer or insulating material was inserted into the space. Hemostasis was achieved using bipolar coagulation, gentle compression, suction, and irrigation, and the field was kept as dry as possible prior to cryoablation. We acknowledge that the surgically created space may not remain air-filled and could subsequently become partially fluid-filled (e.g., serosanguinous fluid), which would alter its thermal properties compared with a pure air gap. In the 0-mm group, only a laminectomy was performed.

### 2.8 Cryoablation procedure

Vertebral cryoablation at T13 was performed using a 1.5-mm-diameter, copper-tipped cryoprobe introduced from the left side into the vertebral body. Cooling was achieved with a nonclinical experimental setup: liquid nitrogen was delivered through a custom syringe–metal wire system connected to the probe (Kobayashi et al., 2024; Annen et al., 2022). A single 10-min freeze cycle commenced once the probe tip temperature reached < −40 °C, a commonly used lethal isotherm for cryoablation (Littrup et al., 2009).

*Probe trajectory and depth control*: Probe placement was standardized using direct anatomic landmarks. After exposing the vertebral body, the cranial and caudal intervertebral discs were visually identified to define the craniocaudal mid-vertebral level. The posterior vertebral wall had been removed during the separation procedure and was therefore unavailable as a landmark. The anterior vertebral wall was palpated with a spatula to establish the anterior boundary. Using the spatula as an anterior reference, a cortical entry hole was created on the lateral vertebral body cortex approximately 2 mm dorsal to the spatula tip (corresponding to one 2-mm burr diameter). The cryoprobe was then advanced perpendicular to the lateral cortex to a fixed insertion depth of 10 mm, targeting the mid-vertebral level under direct visualization while avoiding anterior wall breach.

### 2.9 Temperature and electrophysiological monitoring

All temperatures were monitored continuously, and data were logged every 10 s (GL240-SD; Graphtec Corp., Yokohama, Japan). The intravertebral temperature at the tip of the cryoprobe was monitored using a sheath-type thermocouple. Ventral epidural temperature was measured using a single spatula-type thermocouple positioned at the midline between the ventral dura mater and the preserved posterior longitudinal ligament.

Spinal cord function was assessed by recording CMAPs evoked by epidural spinal cord stimulation. CMAPs were recorded from the bilateral soleus muscles using needle electrodes (NE-110B; Nihon Kohden). Stimulation was delivered via a dura-contacting platinum coil electrode (USY-100-2PMC; Unique Medical) placed on the posterior midline of the dura at the T9 level. Stimulation consisted of a 1 Hz train of five 0.5-ms rectangular pulses (interstimulus interval, 2.0 ms), with intensity set 10% above the threshold for maximal amplitude (2–3 mA range). Signals were acquired with a 10–3000 Hz band-pass filter and averaged over 10 responses (Neuropack Σ electromyography system; Meb-5504, Nihon Kohden). Baseline CMAP amplitude was defined as the mean of three stable responses recorded immediately before freezing (after the separation procedure and just prior to cryoablation). CMAPs were then recorded at 10 min and 30 min after the start of cryoablation. For analysis, CMAP amplitude at each time point was calculated as the mean of bilateral soleus amplitudes. A > 70% decrease from baseline CMAP amplitude was defined as a safety alert, analogous to widely used transcranial motor-evoked potential criteria in spine surgery, with many series adopting approximately 70% and others using a 50–80% range (Tanaka et al., 2011; Park and Hyun, 2015; Dulfer et al., 2024). This alert criterion was pragmatically adapted from mechanical spine-surgery neuromonitoring literature and has not been specifically validated as a threshold for impending thermal injury; therefore, it was used as a practical safety surrogate and not a definitive cutoff. We acknowledge that propofol and nitrous oxide can influence myogenic motor responses; therefore, we used an identical anesthetic regimen and standardized recording conditions were applied across all animals. To ensure internal consistency, baseline CMAP amplitude was defined immediately before freezing (after completion of the separation procedure and immediately prior to cryoablation).

### 2.10 Outcomes

The primary endpoints were the minimum *in vivo* ventral epidural temperature and 30-min CMAP amplitude recovery. Exploratory endpoints included *in vitro* temperatures. CMAP recovery was defined as 100 × (CMAP amplitude at 30 min/baseline CMAP amplitude). Baseline CMAP amplitude was defined as the mean of three stable responses recorded immediately before freezing (after completion of the separation procedure and immediately prior to cryoablation). Predefined exclusion criteria included lack of a stable baseline or recording artifacts. No animals met these criteria, and all datasets were included in the primary analyses.

### 2.11 Ethical approval

All animal experiments were approved by the Institutional Animal Care and Use Committee of Kanazawa University (Approval No. AP-224381). This study was conducted and reported in accordance with the Animal Research: Reporting of In Vivo Experiments (ARRIVE) guidelines; the completed ARRIVE checklist is provided as supplemental material.

### 2.12 Statistical analysis

All statistical analyses were performed using JASP (version 0.95.3). Data from *in vitro* experiments are presented as mean ± standard deviation (SD), whereas data from *in vivo* experiments are presented as both mean ± SD and median [interquartile range, IQR]. Differences among the three groups were assessed using the Kruskal–Wallis test. Post hoc pairwise comparisons were performed using the Dwass–Steel–Critchlow–Fligner (DSCF) test for *in vitro* data and Dunn's test with Holm correction for *in vivo* data. A *P* value of < 0.05 was considered significant. Nonparametric tests were prespecified, given the small sample size and non-Gaussian pilot distributions; therefore, no parametric assumption diagnostics were undertaken.

## 3 Results

### 3.1 *In vitro* temperature results

**Fig. 2:**
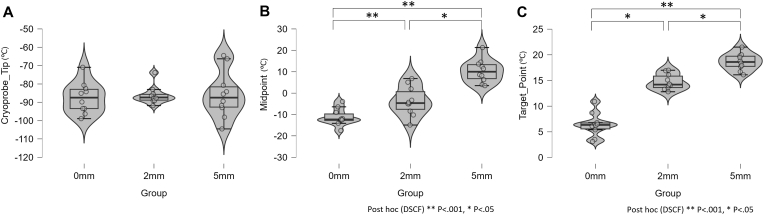
*In vitro* temperature results. Violin and box plots show minimum temperatures at (A) cryoprobe tip, (B) midpoint, and (C) target point for 0-, 2-, and 5-mm groups; individual data points overlaid. Boxes = median and IQR; whiskers = range. Asterisks/brackets denote pairwise significance by DSCF (adjusted): all pairs significant at Target point (5 vs 0, *P* < .001; 5 vs 2, *P* = .002; 0 vs 2, *P* < .001) and at Midpoint (5 vs 0, *P* < .001; 5 vs 2, *P* = .002; 0 vs 2, *P* = .034).

The *in vitro* phantom experiments demonstrated a significant distance-dependent thermal-insulating effect (Fig. 2, Table 1). Detailed per-trial data are provided in Supplementary Table 1. At the target point, the mean ± SD minimum temperatures were 6.7 ± 2.3 °C for the 0-mm group, 15.2 ± 1.4 °C for the 2-mm group, and 19.2 ± 1.4 °C for the 5-mm group. The Kruskal–Wallis test showed a statistically significant difference among the three groups (H(2) = 24.7, *P* < .001). Post hoc analysis using the DSCF test revealed significant differences between all pairs: 5-mm vs 0-mm (*P* < .001), 5-mm vs 2-mm (*P* = .002), and 0-mm vs 2-mm (*P* < .001). A similar significant effect was observed at the midpoint (H(2) = 20.7, *P* < .001), with post hoc tests confirming significant differences between all pairs (*P* ≤ .034). In contrast, there was no significant difference in the minimum temperatures recorded at the cryoprobe tip among the three groups (mean ± SD: −87.0 ± 8.7 °C for 0-mm, −85.9 ± 6.1 °C for 2-mm, and −85.2 ± 13.4 °C for 5-mm; Kruskal–Wallis H(2) = 0.209, *P* = .901), indicating comparable probe performance across conditions.

**Table 1:** *In vitro* temperature results at the end of the 10-min freeze cycle.

Measurement point	0-mm group (n = 10)	2-mm group (n = 10)	5-mm group (n = 10)
Cryoprobe tip (°C)	−87.0 ± 8.7	−85.9 ± 6.1	−85.2 ± 13.4
Midpoint (°C)	−11.0 ± 4.1	−4.1 ± 7.8	11.1 ± 6.0
Target point (°C)	6.7 ± 2.3	15.2 ± 1.4	19.2 ± 1.4

Data are mean ± SD. Kruskal–Wallis: target point H(2) = 24.7, *P* < .001; Midpoint H(2) = 20.7, *P* < .001; Cryoprobe tip H(2) = 0.209, *P* = .901. Post hoc (DSCF): all pairs were significant at the target point (5 vs 0, *P* < .001; 5 vs 2, *P* = .002; 0 vs 2, *P* < .001) and at the midpoint (5 vs 0, *P* < .001; 5 vs 2, *P* = .002; 0 vs 2, *P* = .034).

### 3.2 *In vivo* temperature results

**Fig. 3:**
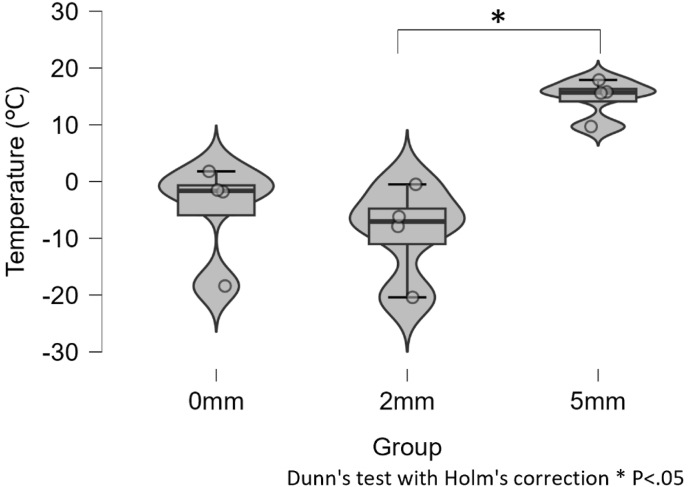
*In vivo* minimum epidural temperature. Violin and box plots with individual animals show group distributions. Overall Kruskal–Wallis H(2) = 7.731, *P* = .021; Dunn–Holm post hoc: 2 vs 5 mm significant (*P* = .024), 0 vs 5 mm: not statistically significant (*P* = .079); 0 vs 2 mm: not significant (*P* = .556).

The minimum epidural temperature differed significantly among the three separation groups (Kruskal–Wallis, H(2) = 7.731, *P* = .021). The median [IQR] minimum epidural temperature was −1.65 °C [−13.25 to 0.98] in the 0-mm group, −7.05 °C [−17.28 to −2.43] in the 2-mm group, and 15.7 °C [9.7 to 17.9] in the 5-mm group (Fig. 3; Table 2). Individual animal data are provided in Supplementary Table 2. Post hoc Dunn's tests with Holm correction showed a statistically significant difference between the 2-mm and 5-mm groups (*P* = .024); differences between 0-mm vs 5-mm (*P* = .079) and 0-mm vs 2-mm (*P* = .556) were not significant. Individual ventral epidural temperature time-series for each animal are shown in Supplementary Fig. S1.

**Table 2:** *In vivo* minimum epidural temperature and CMAP amplitude recovery.

Parameter	0-mm group (n = 4)	2-mm group (n = 4)	5-mm group (n = 4)
**Minimum epidural temperature (°C)**			
***Median [IQR]***	**−1.65 [−13.25 to 0.98]**	**−7.05 [−17.28 to −2.43]**	**15.7 [9.7 to 17.9]**
*Mean ± SD*	−5.0 ± 9.8	−8.8 ± 8.4	14.5 ± 4.2
**CMAP recovery at 30 min (%)**			
***Median [IQR]***	**38.2 [11.1 to 58.9]**	**13.8 [3.85 to 37.55]**	**87.15 [82.95 to 91.8]**
*Mean ± SD*	35.0 ± 29.5	20.7 ± 24.4	87.4 ± 6.4

Data are presented as median [interquartile range, IQR] or mean ± SD. Kruskal–Wallis: minimum epidural temperature H(2) = 7.731, *P* = .021; CMAP recovery H(2) = 7.652, *P* = .022. Post hoc (Dunn with Holm): 2 vs 5 mm significant for both endpoints (*P* = .024 and 0.028, respectively); 0 vs 5 mm trend (*P* = .079 and 0.069); 0 vs 2 mm not significant (*P* = .556 and 0.623). CMAP = compound muscle action potential.

### 3.3 *In vivo* CMAP recovery

**Fig. 4:**
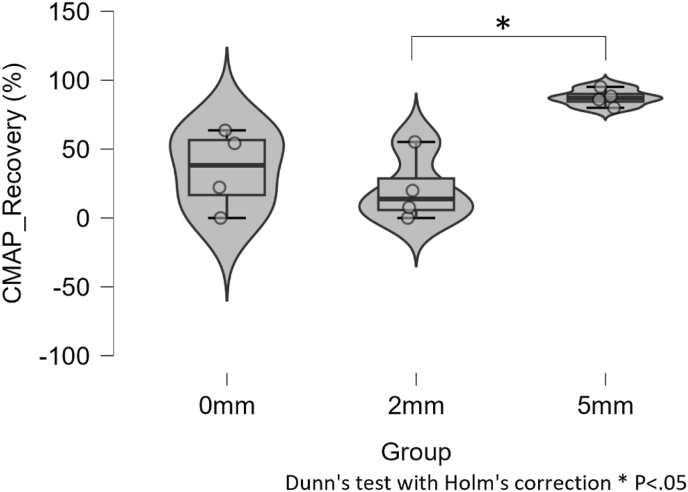
*In vivo* CMAP amplitude recovery at 30 min post-cryoablation. Violin and box plots with individual animals. Overall Kruskal–Wallis H(2) = 7.652, *P* = .022; Dunn–Holm post hoc: 2 vs 5 mm significant (*P* = .028); 0 vs 5 mm trend (*P* = .069); 0 vs 2 mm: not significant (*P* = .623). CMAP = compound muscle action potential.

Baseline was defined immediately before freezing (after completion of the separation procedure). Relative to this pre-freeze baseline, CMAP amplitudes at 10 min after the start of cryoablation (end of the freeze phase) were markedly reduced across animals, often approaching near-abolition (Supplementary Fig. S2). Recovery of CMAP amplitude at 30 min after the start of cryoablation differed significantly among the three separation groups (Kruskal–Wallis H(2) = 7.652, *P* = .022) (Fig. 4; Table 2). The median [IQR] recovery rate was 38.2% [11.1 to 58.9] for the 0-mm group, 13.8% [3.85 to 37.55] for the 2-mm group, and 87.15% [82.95 to 91.8] for the 5-mm group. Dunn–Holm post hoc testing showed a significant difference between 2-mm and 5-mm (*P* = .028). The comparisons for 0-mm vs 5-mm (*P* = .069) and 0-mm vs 2-mm (*P* = .623) were not statistically significant, possibly reflecting the limited power of this exploratory study. Individual animal CMAP amplitude trajectories at baseline/pre-freeze, 10 min, and 30 min are shown in Supplementary Fig. S2, with individual summary values provided in Supplementary Table 2.

## 4 Discussion

This proof-of-concept study explored the association between surgically created epidural separation distance and short-term thermal and neurophysiological changes during vertebral cryoablation. In the *in vitro* phantom, increasing the separation distance was associated with higher minimum temperatures at the target point, consistent with a distance-dependent thermal gradient under controlled conditions. *In vivo*, greater separation distance was associated with higher minimum temperatures at the ventral midline epidural point and with higher 30-min CMAP amplitude recovery, with the 5-mm group showing the most favorable outcomes within the tested range. These observations indicate that, in this canine model and within the tested range, larger epidural separation is accompanied by warmer temperatures at the measured epidural location and better short-term neurophysiological recovery. Because our temperature measurements represent a single-point ventral midline epidural recording and this was a terminal non-survival study without histology, these findings should be interpreted as hypothesis-generating rather than as evidence of spinal cord injury prevention.

The approximately 4 °C epidural temperature benchmark used in this study was drawn from prior work using a similar canine model by Kobayashi et al. (2024). In that study, epidural temperatures that remained at or above 4 °C were associated with transient, recoverable neurophysiological changes, whereas lower epidural temperatures were associated with histological injury and/or persistent deficits in their survival model. In contrast, minimum epidural temperatures between 1 °C and 0 °C resulted in histologically confirmed cryogenic injury despite functional recovery, and temperatures of −8 °C or lower led to both persistent CMAP suppression and permanent motor deficits. Accordingly, we used 4 °C as a preclinical reference point to contextualize our measured ventral epidural temperatures, not as a definitive safety threshold. Moreover, thresholds for irreversible injury may differ across models, anatomical contexts, and devices and therefore cannot be inferred directly from our point measurements.

A central question for this work was whether separation distance is associated with the minimum ventral epidural temperature during vertebral cryoablation. The *in vitro* and *in vivo* results were directionally consistent, and cryoprobe tip temperatures were similar across conditions, arguing against differences in delivered cooling power at the probe tip as the primary explanation for between-group differences at the epidural measurement site. However, given the non-randomized, non-blinded design and the potential for geometric and day/order effects, we interpret the observed distance–temperature pattern as consistent with a distance-dependent insulating effect, rather than evidence that separation distance alone determines epidural temperature *in vivo*.

The functional findings were also consistent with the thermal observations at the measured ventral epidural point. CMAP recovery at 30 min differed among groups, and post hoc comparisons showed higher recovery in the 5-mm group than in the 2-mm group. These results are compatible with established physiological effects of cooling on neural conduction (Algafly and George, 2007; Herrera et al., 2010) and with general principles of cryoinjury related to nadir temperature and time at temperature (Erinjeri and Clark, 2010). Nevertheless, CMAP measurements were limited to predefined timepoints and do not establish a mechanistic link between epidural temperature and tissue injury. Accordingly, we describe the CMAP findings as short-term neurophysiological changes associated with separation distance, rather than as evidence of neuroprotection.

The 2-mm group showed numerically less favorable epidural temperature and CMAP recovery than the 0-mm group, although no statistically significant difference was detected. Given the exploratory design, limited power, and potential day/order and measurement biases, this finding remains inconclusive.

Physical separation of critical structures is used in other ablation settings to reduce collateral thermal exposure, including displacement techniques such as hydrodissection or gas insufflation (Tsoumakidou et al., 2011; Seager et al., 2021; Adebanjo et al., 2024). Our findings are consistent with the possibility that a similar distance-dependent thermoprotective principle may operate in the epidural space. However, because the epidural environment is moist and dynamic, the surgically created separation should be considered a “separation space” rather than a persistent “air gap.” The composition of this space may change over time and may become partially fluid-filled (e.g., blood, cerebrospinal fluid, or irrigation fluid), which would increase effective thermal conductivity and permit convection compared with air.

Within the tested range, these data support the hypothesis that planned epidural separation may be associated with warmer ventral epidural temperatures and better short-term CMAP recovery during vertebral cryoablation in this model. They do not establish a clinical strategy. Future work should test this hypothesis in clinically relevant scenarios, including models with epidural tumor and/or cord compression, ideally using survival designs with histology and long-term functional outcomes. Such studies should also quantify geometric factors (e.g., probe trajectory and distance to the posterior vertebral wall), characterize the composition of the separation space over time, and evaluate whether real-time monitoring and guidance technologies improve the reproducibility of separation targets and thermal margins.

### 4.1 Major methodological limitations

This study has some limitations that should be considered when interpreting the findings. First, the exploratory design involved a modest sample size (n = 4 per group) without a formal power calculation, making the study susceptible to Type II errors and rendering non-significant comparisons inconclusive. More critically, the study was neither randomized nor blinded. A fixed daily sequence was used instead of randomization, introducing a risk of order effects. Furthermore, the lack of blinding for surgeons, operators, and data analysts increases the risk of measurement and interpretation bias. Although we considered a separate, blinded data analyst, the distinct thermal and neurophysiological patterns limited the feasibility of effective masking. These design features represent a threat to internal validity, and the results should be interpreted cautiously.

Second, external validity was constrained by the model. The *in vivo* experiments were performed in an intact spinal canal without epidural tumor or cord compression; therefore, cerebrospinal fluid circulation was likely preserved. This may have attenuated epidural cooling compared with clinical scenarios involving severe cord compression or blocked cerebrospinal fluid flow. Accordingly, the separation distances tested here, including 5 mm, should not be interpreted as universally safe distances for metastatic spine disease. The canine T13 model may not reflect human anatomic variability, bone quality, or perfusion conditions encountered clinically. Additionally, the *in vitro* phantom at 25 °C likely overestimates safety margins relative to perfused tissue.

Third, several technical aspects limit direct clinical translation. Cooling was delivered using a nonclinical, custom liquid-nitrogen system, which differs from clinical argon-based Joule–Thomson probes in its cooling dynamics and ice-ball geometry. Thus, the specific distances tested should be interpreted as illustrating the principle of distance dependence rather than as device-agnostic clinical thresholds. Epidural temperature was sampled at a single ventral midline location and therefore does not capture three-dimensional thermal gradients around the cord; the reported temperatures should be interpreted as point measurements rather than as the minimum temperature experienced by the spinal cord parenchyma. The separation space was not instrumented to verify whether it remained air-filled or became fluid-filled during cryoablation. Because fluid filling would alter effective thermal conductivity and enable convection, the *in vivo* data should be interpreted as reflecting the net effect of a surgically created separation space under the experimental conditions, rather than the insulating effect of a stable air layer. Although anesthesia was standardized, propofol and nitrous oxide can affect myogenic motor responses; therefore, residual anesthetic-related confounding cannot be completely excluded. The >70% CMAP decrease threshold used as a safety alert was extrapolated from literature on mechanical spinal cord injury, and its validity for predicting irreversible thermal injury is not established. Finally, histology and long-term behavioral or functional outcomes were not obtained because the *in vivo* study was conducted as a terminal non-survival procedure.

Despite these limitations, this proof-of-concept study demonstrates that epidural separation distance was associated with ventral midline epidural temperature and short-term CMAP recovery during vertebral cryoablation. These findings should be interpreted as hypothesis-generating and require validation in clinically relevant, preferably survival, models with histology and longer-term functional outcomes.

## 5 Conclusions

In this pilot canine model, a 5-mm epidural separation was associated with higher minimum temperatures at the measured ventral midline epidural measurement point and with better short-term CMAP amplitude recovery compared with smaller separations during vertebral cryoablation. These proof-of-concept data support the principle of distance-dependent associations between separation distance and measured thermal and neurophysiological endpoints, and provide a rationale for future studies to validate separation-augmented cryoablation in clinically relevant, preferably survival, models with histological and longer-term functional outcomes.

## Ethics approval

All animal procedures were approved by the Institutional Animal Care and Use Committee of Kanazawa University (Approval No. AP-224381) and conducted in accordance with institutional and national guidelines.

## Consent for publication

Not applicable.

## Availability of data and materials

The datasets generated and analyzed during the current study are available from the corresponding author on reasonable request.

## Funding

This work was supported by 10.13039/501100001691JSPS KAKENHI (Grant No. 24K15823). The funding body had no role in the design of the study; in the collection, analysis, and interpretation of data; in the writing of the manuscript; or in the decision to submit the article for publication.

## Declaration of competing interest

The authors declare the following financial interests/personal relationships which may be considered as potential competing interests: Takaaki Uto reports financial support was provided by Japan Society for the Promotion of Science. If there are other authors, they declare that they have no known competing financial interests or personal relationships that could have appeared to influence the work reported in this paper.

## Appendix A Supplementary data

Multimedia component 2 Multimedia component 2

Fig. S1 Individual ventral epidural temperature time-series for each animal during vertebral cryoablation, stratified by epidural separation distance (0, 2, and 5 mm). The shaded area indicates the 10-min freeze phase (0–600 s). Fig. S2. Individual compound muscle action potential (CMAP) amplitude trajectories (% of baseline) at baseline (0 s), end of freezing (600 s), and 30 min after the start of cryoablation (1800 s), stratified by epidural separation distance (0, 2, and 5 mm). The shaded area indicates the 10-min freeze phase (0–600 s) Multimedia component. 1
