# Supplementary material for: Spinal cord protection by epidural separation during vertebral cryoablation for metastatic spine disease: A proof-of-concept preclinical study
Source: Brain Spine. 2026 Feb 13;6:105975. doi: 10.1016/j.bas.2026.105975 (PMC12925502; doi:10.1016/j.bas.2026.105975)
Supplement: Multimedia component 1 [file mmc1.pdf]

Supplemental Data

Supplementary Table 1. Per-Trial Data (*In Vitro*)

Trial ID	Gap (mm)	Tip Min (°C)	Midpoint Min (°C)	Target Min (°C)	Room Temp (°C)
01	0	-70.9	-3.9	10.9	24.3
02	0	-98.9	-6.4	6.1	25.8
03	0	-84.2	-17.5	3.5	27.8
04	0	-93.4	-12.8	6.5	26.3
05	0	-93.4	-9.0	8.7	27.2
06	0	-82.5	-14.1	3.1	27.3
07	0	-96.3	-12.5	6.8	25.7
08	0	-89.8	-11.9	6.2	25.7
09	0	-85.1	-12.1	6.5	25.8
10	0	-80.7	-12.5	5.4	26.1
11	2	-73.8	6.8	16.9	24.2
12	2	-91.8	-5.0	14.1	25.0
13	2	-87.0	5.1	17.0	25.5
14	2	-85.4	-14.9	13.5	26.0
15	2	-86.5	-10.2	16.1	27.0
16	2	-89.1	-5.0	15.1	27.3
17	2	-87.9	-4.3	14.0	27.2
18	2	-83.0	-3.3	13.3	24.9
19	2	-89.1	2.0	14.3	27.0
20	2	-87.7	-8.4	12.8	27.6
21	5	-64.5	3.5	17.9	25.8
22	5	-66.3	12.4	19.8	24.2
23	5	-92.9	21.3	21.5	25.6
24	5	-84.2	14.5	20.0	25.6
25	5	-85.5	11.4	18.8	25.3
26	5	-98.1	13.6	19.5	26.2
27	5	-104.5	8.6	17.7	25.6
28	5	-89.4	8.2	18.4	25.6
29	5	-91.7	4.0	16.7	26.4
30	5	-80.7	6.3	16.1	27.2

Supplementary Table 2. Individual Animal Data (*In Vivo*)

Dog ID	Group	Min Epidural Temp (°C)	CMAP Recovery (%)
01	0 mm	-18.4	22.2
02	0 mm	-1.8	63.6
03	0 mm	-1.5	54.2
04	0 mm	1.8	0.0
05	2 mm	-0.5	55.2
06	2 mm	-20.4	0.0
07	2 mm	-7.9	19.9
08	2 mm	-6.2	7.7
09	5 mm	9.7	80.0
10	5 mm	15.8	95.2
11	5 mm	17.9	88.4
12	5 mm	14.6	85.9
