# Supplementary figures and images for: Spinal cord protection by epidural separation during vertebral cryoablation for metastatic spine disease: A proof-of-concept preclinical study
Source: Brain Spine. 2026 Feb 13;6:105975. doi: 10.1016/j.bas.2026.105975 (PMC12925502; doi:10.1016/j.bas.2026.105975)

**Fig. S1.**
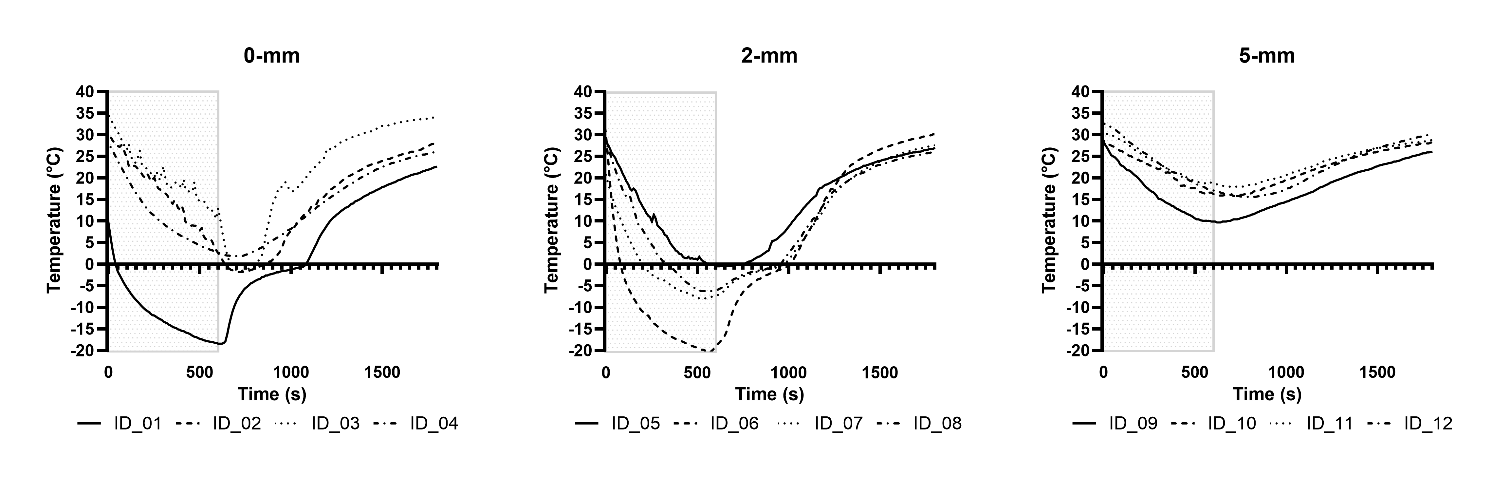


**Fig. S2.**


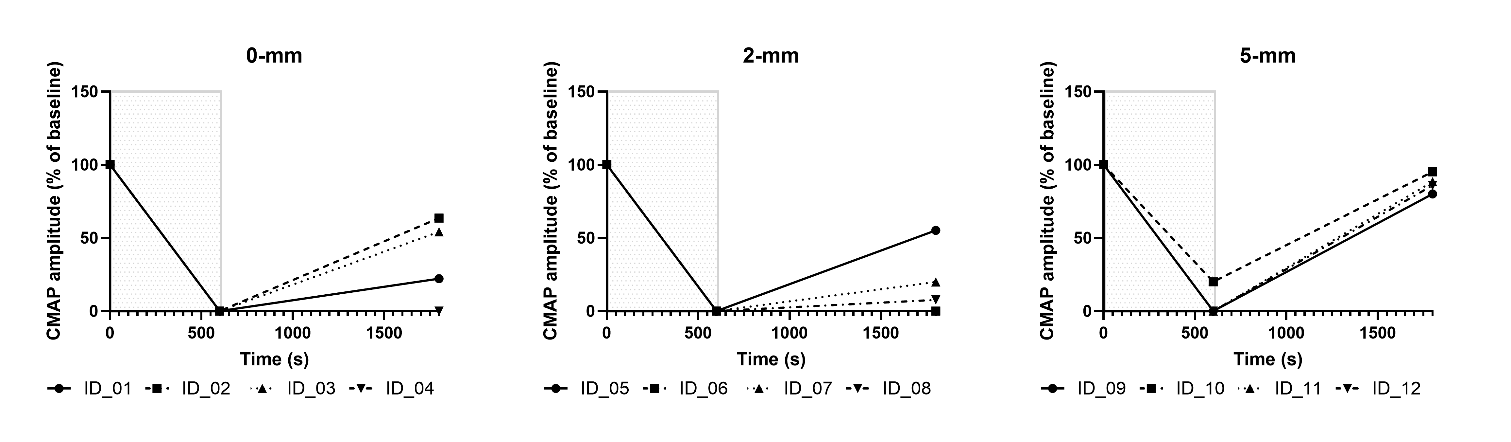

Supplement: Supplementary file 3 — Fig. S1 Individual ventral epidural temperature time-series for each animal during vertebral cryoablation, stratified by epidural separation distance (0, 2, and 5 mm). The shaded area indicates the 10-min freeze phase (0–600 s). Fig. S2. Individual compound muscle action potential (CMAP) amplitude trajectories (% of baseline) at baseline (0 s), end of freezing (600 s), and 30 min after the start of cryoablation (1800 s), stratified by epidural separation distance (0, 2, and 5 mm). The shaded area indicates the 10-min freeze phase (0–600 s) Multimedia component. 1 [file mmc3.docx]
